# Precision, prognosis, and clinical performance of rounded and trabecular segmentation of cine cardiovascular magnetic resonance

**DOI:** 10.1016/j.jocmr.2025.102014

**Published:** 2025-11-25

**Authors:** George Joy, James C. Moon, Karan Punjabi, Mohammed Alzahir, Jessica Artico, Hunain Shiwani, Iain Pierce, Anish Bhuva, Dhruv Thakur, Hui Xue, Peter Kellman, Erik Schelbert, Thomas A. Treibel, Charlotte Manisty, Rhodri H. Davies

**Affiliations:** a Institute of Cardiovascular Sciences, University College London, London, UK; b Barts Heart Centre, St Bartholomew’s Hospital, London, UK; c Cardiovascular Clinical and Academic Group, City St George’s University of London, London, UK; d Health Futures, Microsoft Research, Redmond, Washington, USA; e National Heart, Lung, and Blood Institute, National Institutes of Health, Bethesda, Maryland, USA; f Allina Health Minneapolis Heart Institute at United Hospital, Saint Paul, Minnesota, USA; g University of Pittsburgh Medical Center, Pittsburgh, Pennsylvania, USA

**Keywords:** Cardiac MRI, Segmentation, Artificial intelligence, Trabecular, Rounded

## Abstract

**Background:**

Measurements of cardiac size and function drive clinical decisions. Left ventricular (LV) metrics can be derived from cardiac MR images by delineating the blood pool and myocardium, by either drawing a *rounded* contour to approximate the compacted myocardial border, or by delineating the papillary muscles and trabeculae (*trabecular* segmentation). There is no consensus as to which is best, particularly in the emergent AI era. We developed machine-learning (ML) approaches for both and compared them for clinically important metrics (error rate, precision, and prognosis).

**Methods:**

Separate ML models were developed for *rounded* and *trabecular* segmentation, using U-net models trained on 1923 subjects (mixed pathology, multiple scanners, multiple centers). Blood and myocardial volumes for each segmentation method were compared on 4118 healthy UK biobank subjects. Model segmentation quality was evaluated subjectively on a real-world clinical dataset of 1594 consecutive CMR scans, with all scans included regardless of image quality and artifacts. Scan-rescan precision was measured on a multi-center, multi-disease dataset of 109 subjects scanned twice and compared to human performance. Finally, prognostication ability was evaluated on 1215 clinical patients, using a primary outcome of all-cause mortality and hospitalization with heart failure.

**Results:**

Error rates (where a human disagreed by >1 mL) were the same, occurring in 0.6% (184/29680) of images and 3.6% (60/1594) of patients. In health, the mean EF was 4% higher for trabecular vs rounded segmentation. On test-retest data, there was no difference between rounded and trabecular ML models for precision, apart from end-diastolic and end-systolic volume, which was better for rounded segmentations. ML rounded and trabecular precision exceeded clinician performance for EF. There were marginal differences in prognostication between rounded and trabecular models.

**Conclusion:**

We developed an automated method for annotating papillary muscles and trabeculae from cardiac MR images with low error rates. We found higher precision than clinicians in ejection fraction. There was similar precision and prognostication to an ML rounded model with similarly low error rates. Findings support the feasibility of automated trabecular segmentation in clinical care and clinical trials.

## Graphical abstract

**Figure:**
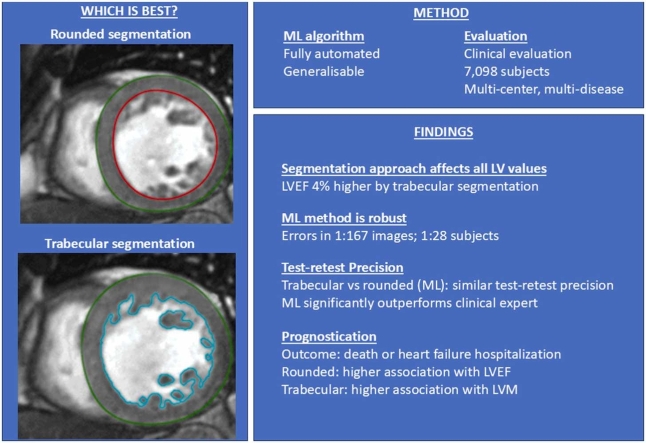

## 1 Introduction

**Fig. 1:**
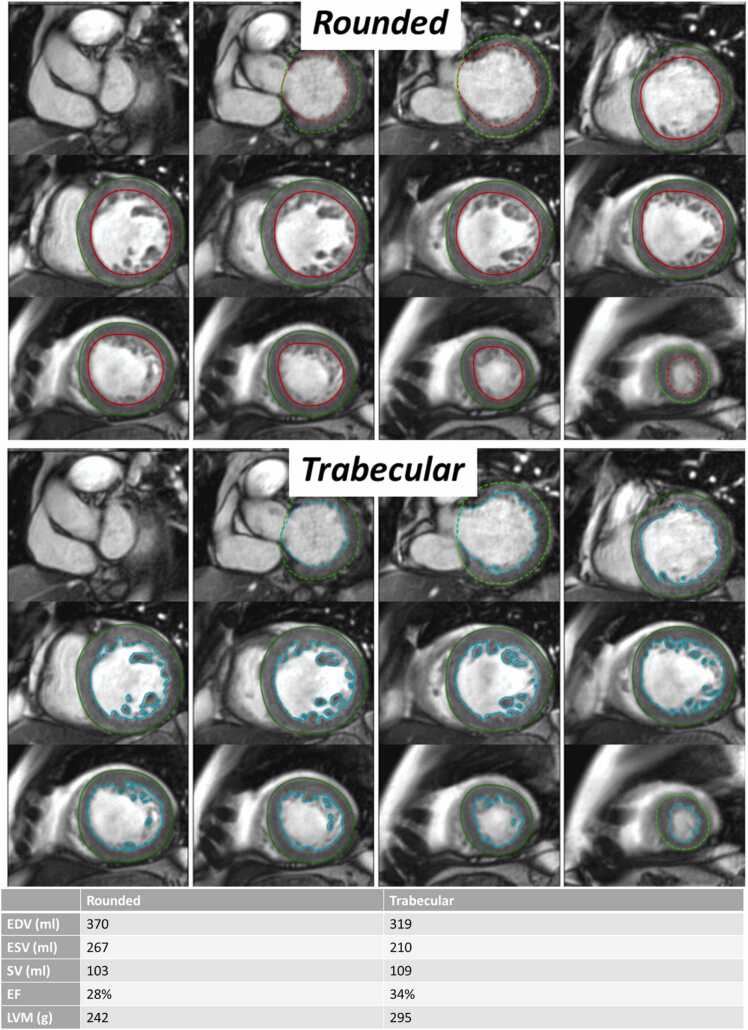
Illustration of rounded (top) and trabecular (bottom) segmentation approaches in a patient with non-ischemic cardiomyopathy (diastole). A rounded contour is used to approximate the endocardial border between compacted and non-compacted myocardium. Trabecular segmentation involves separately delineating all myocardium, including the papillary muscles and trabeculae. The table at the bottom illustrates how a smaller EDV leads to a larger EF despite a similar SV. Data corresponds to LV metrics for the examplar case in the Illustration. *EDV* end diastolic volume, *EF* ejection fraction, *SV* stroke volume

Many clinical decisions in cardiology are driven by measures of left ventricular (LV) structure and function. Measures such as end diastolic volume (EDV), end systolic volume (ESV), left ventricular ejection fraction (EF), and LV myocardial mass (LVM) can be derived from cardiovascular MR (CMR) cine images by delineating (“segmenting”) the myocardium and LV blood pool. The quickest way is to draw a rounded contour to approximate the LV endocardium between compacted and non-compacted myocardium (Fig. 1). However, this approach treats papillary muscles and trabeculae as part of the blood pool, which is anatomically incorrect and hence limits accuracy. Trabecular morphology is an important determinant of cardiac performance [1] and detailed segmentation of the papillary muscles and trabeculae (Fig. 1) may provide additional insights, particularly into diseases characterized by myocardial infiltration or myocyte hypertrophy, such as cardiac amyloidosis and hypertrophic cardiomyopathy (HCM) [2]. There is no consensus on whether *rounded* or *trabecular* segmentation is best.

Advances in machine learning (ML) have led to robust algorithms for segmenting the LV blood pool and myocardium [3], [4], [5]. However, most ML methods continue to use rounded segmentations because of lack of detailed annotations of the trabeculae and papillary muscles on which to train the model. Recent work has shown that development of ML trabecular segmentation is feasible [6], [7]. We have previously described an ML approach that produced a fully automated, quick, generalizable (all patients, all scanners), and robust model that improved on clinician precision of LV parameters and wall thickness [3], [8]. We sought to build on this approach to build a fully automated detailed segmentation of papillary muscles and trabeculae from CMR cine images.

The ML trabecular model is evaluated on several external, independent datasets and compared to a clinician and to an ML model trained on rounded segmentations. Differences in LV metrics produced by the rounded and trabecular models are compared on a set of healthy subjects. Qualitative evaluation of segmentation is performed on a large prospective cohort of successive patients referred for a cardiac MR scan as part of their clinical care. Test-retest precision is measured on a multicentre dataset containing multiple pathologies. Finally, prognostication ability is evaluated on a further external clinical dataset.

## 2 Method

All data were acquired from subjects who had previously participated in clinical studies with formal written consent and either national (Research Ethics approval IDs: 141186, 294495, 07/H0715/101) or local ethical approval and performed in keeping with the principles outlined in the Helsinki declaration.

### 2.1 ML approach

We follow the ML approach that we have previously described [3], but summarize the approach below, and describe any changes made for this work. Subjects were recruited via written informed consent to clinical studies, all with approval from the local research ethics committees. Further details of the data can be found in [3].

#### 2.1.1 Training data

Standard cardiac CMR cine images (short axis stack and three long axis imaging views) were acquired from 1923 patients, at two field strengths (1.5 and 3T), using three CMR manufacturers (Siemens Healthineers, Erlangen, Germany; Philips Healthcare, Best, the Netherlands; General Electric Healthcare, Chicago, Illinois, USA), 10 scanner models, across 13 institutions in 3 countries, were used for model training, as described previously [3]. Multiple cardiac phenotypes were included in the training data, including healthy volunteers, athletes (as a model of physiological adaptation) and several disease cohorts (e.g., aortic stenosis, amyloid, Fabry, hypertension, hypertrophic cardiomyopathy, myocardial infarction). Some iterative improvements have been made to the training data annotations since the original publication.

Manual segmentation of training images was performed using CVI42 (version 5.3.8; Circle Cardiovascular Imaging, Calgary, Alberta, Canada). Rounded segmentations were created by three clinicians with the semi-automated threshold tool with the “smoothed contour” option enabled and post hoc freehand correction used as necessary. Initial trabecular segmentations which included trabecular tissue and papillary muscles in LV myocardium, were made using the threshold tool in cvi42 and refined manually by an experienced clinical radiologist (MA).

#### 2.1.2 Image Pre-processing

Standard short-axis cine images were spatially normalized as described previously [3]. In brief, images were translated so that the intersection point of the short-axis, 4-chamber, and 2-chamber images was at the image center; images were scaled to achieve an in-plane voxel resolution of 1 mm^2^; and rotated so that the intersection line of the 2-chamber with short-axis image was parallel to the y-axis. Each image histogram was clipped at the 99th centile and normalized to lie in the range [0,1] and all images were center-cropped or padded to 160 × 160 pixels.

#### 2.1.3 Mitral annular plane definition

The base of the myocardium (the transition between ventricle and atrium) is difficult to define on short-axis images. We therefore employ the 2-chamber and 4-chamber cine images to define the mitral annulus, represented by 2 points at the intersection of the mitral valve and myocardium on the 2- and 4-chamber images [3].

#### 2.1.4 Convolutional neural network architecture and training

A Unet architecture was used with dilated (Atrous) convolutions and batch normalization. The Unet configuration was based on the best-performing structure that we found empirically in previous work [3]. In brief: the encoder consisted of layers of dilated 3×3 convolution, batch normalization, and ReLU, with a 2×2 maxpool between layers. The decoder blocks consisted of dilated 3×3 convolution, batch normalization, and ReLU with 2×2 transposed convolution in between. There were 4 layers in the encoder, 4 layers in the decoder and a bottleneck layer. 32 features were used in the first layer, and this was doubled at each layer in the encoder and halved at each layer in the decoder, and standard Unet skip layer concatenation was performed.

#### 2.1.5 Adaptation for trabecular segmentation

The trabecular model was trained using the same images, U-net structure, and hyperparameters as the rounded segmentation model. The same mitral annular plane was used to clip both rounded and trabecular segmentations, as described above. We first attempted to train the trabecular model in an identical manner to the rounded segmentation model, but better performance was achieved by first using the rounded segmentation model to define a bounding box around the LV myocardium, and resampling to 80×80 pixels. The trabecular model was then trained on the 80×80 images—see supplementary Figure 1 for illustration.

### 2.2 Evaluation

#### 2.2.1 Subjective assessment of segmentation quality

Quality of image segmentation was evaluated by an experienced cardiac radiologist (M.A.) and cardiologist (J.A.) on subjects scanned during a 12-week period at Barts Heart Centre (3 dedicated cardiac magnets, 2 x iemens Aera 1.5T, 1 x Siemens Prisma 3T). All successive patients referred as part of their clinical care were included, regardless of image quality and presence of artifact. Exclusion criteria were congenital heart disease, incomplete scans, or real-time cine imaging.

**Fig. 2:**
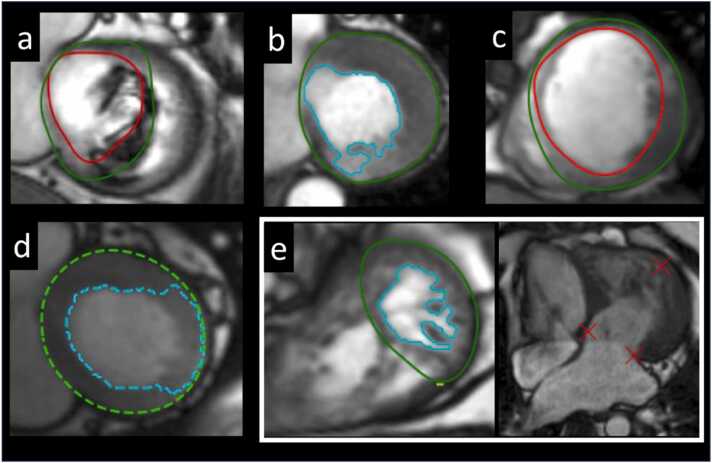
Examples of significant model errors (a) artifact from a prosthetic valve causing mis-segmentation of the rounded contour, (b) mis-segmentation by trabecular model with “leakage” of the contour into the compacted myocardium in the basal inferior wall (c) rounded model mis-segmentation of the apical septum (d) trabecular model mis-segmentation of the lateral wall and (e) mis-segmentation due to extreme anatomy: LV apex is aneurysmal (see 4-chamber image, right) and the trabecular model has only segmented half of the LV. *LV* left ventricular

Errors were considered clinically significant if they were subjectively assessed to affect >1 mL of blood or myocardium, and coded by location (apical, mid, or basal slice), and nature of error (mis-segmentation, severe artifact, or extreme pathology)—see Fig. 2 for examples. Due to the subjective nature of error detection by expert observers, we performed a separate analysis of 533 patient scans to test agreement in error detection between two blinded observers.

#### 2.2.2 Differences in LV metrics due to segmentation approach

To investigate the difference in values of LV metrics due to segmentation approach (rounded vs trabecular), we used a healthy subset of 4118 subjects identified from the imaging substudy of the UK biobank (project number 71702) [9]. Subjects were considered healthy if free from hypertension, diabetes, hypercholesterolemia, peripheral vascular disease, coronary artery disease, and cerebrovascular disease and not taking any regular medications.

#### 2.2.3 Scan-rescan precision

Scan-rescan precision was calculated on a specially acquired dataset (available from www.thevolumesresource.com), consisting of 109 subjects scanned twice within a short timeframe where change between scans was considered unlikely (96% [105/109] of subjects re-scanned within 1 week) [10]. One subject was removed from the original data because it was found to be a duplicate. Data were collected from 5 different institutions at 2 field strengths, containing multiple pathologies: 32 cases of myocardial infarction, 17 left ventricular hypertrophy, 17 non-ischemic cardiomyopathy, 8 cardio-oncology patients, 5 chronic kidney disease, and 30 healthy volunteers. As a benchmark for human precision, the scan-rescan images were also annotated by an experienced clinician with 20 years’ CMR experience, using the semi-automated threshold tool in cvi42 (version 5.3.8, Circle Cardiovascular Imaging) and rounded contours with manual freehand correction as needed [10]. The paired scans were separated and analyzed independently with different study identifiers, to ensure that the human reader was blinded to the results from the paired studies.

#### 2.2.4 Correlation with clinical outcomes

Ability to predict adverse clinical outcomes was measured using a single-center multi-disease cohort. 1215 successive patients undergoing CMR as part of their clinical care were recruited from the University of Pittsburgh Medical Center. Primary outcome was a composite of all-cause mortality and hospitalization with heart failure and occurred in 294/1215 (24%) patients over a 5.5-year follow-up period.

### 2.3 Statistical analysis

Statistical analysis was performed in R (R Foundation for Statistical Computing, Vienna, Austria; www.R-project.org). A p-value of less than 0.05 was considered statistically significant. Data that follow a normal distribution are presented as mean (standard deviation), with non-normally distributed data reported as median (interquartile range). Categorical variables are presented as frequencies and percentages. To compare characteristics across groups, the Student's t-test or the Mann-Whitney U test was used for continuous variables depending on their distribution.

Interobserver agreement for both rounded and trabecular ML segmentation error detection was assessed using weighted Cohen’s kappa for two experienced blinded reviewers. This index quantifies the agreement among two observers evaluating data samples among ordinal categories. The Cohen’s kappa ranges from 0 to 1, with 0 indicating no agreement and 1 indicating perfect agreement.

In the UK-biobank cohort, after confirming a normal distribution using visual inspection of box-plots and Q-Q plots, reference intervals were established using the mean ± 2 standard deviations, in keeping with other reference ranges [11].

Scan–rescan precision was quantified using the coefficient of variation (CoV), calculated by the root mean squared method, with 10,000 bootstrap samples to estimate standard errors and confidence intervals [12]. Multiple testing correction with Bonferroni was applied for the primary precision comparisons (ML-trabecular vs ML-rounded, ML-trabecular vs clinician, and ML-rounded vs clinician) across all five LV parameters (EDV, ESV, SV, LVM, EF), giving an adjusted significance threshold of p<0.0033. The standard error of measurement (SEM) was derived from within-subject variance, and the minimal detectable change (MDC) was calculated as 1.96 × SEM × √2 [13], defining a 95% confidence interval of no change. Intraclass correlation coefficients (ICC [1], [2]) were obtained from a two-way random effects model. For sample size justification, a paired t-test assuming a standard deviation of paired differences of 3% demonstrated that 95 subjects would provide 90% power (α = 0.05) to detect a 1% difference in EF precision; 109 subjects were tested to allow redundancy.

Univariable Cox proportional hazards (PH) models were fitted to compare the prognostic value of ML-trabecular derived left ventricular (LV) volume metrics with those derived from rounded segmentation (e.g., ML trabecular EF vs ML smooth EF). Likelihood ratio (LR) tests were used for pairwise comparisons of individual LV volumes parameters (e.g., ML rounded EF vs ML Trabecular EF) and **χ**^**2**^ model fits were obtained; higher **χ**^**2**^ indicating a superior model fit. Akaike Information Criteria (AIC) were also derived; a lower AIC with a reduction of >4 indicated superior model fit and prediction [14]. C-indices were compared with higher values suggestive of greater discrimination (cut-off of 0.05 indicating significantly greater discrimination). Proportional hazards were assessed using Schoenfeld residuals with the global test from cox.zph with no Cox-model violating the proportional hazards assumption. All statistical analysis was performed in R. The survival R-package was used for Cox PH Models [15]. We further tested how many patients were reclassified across clinically relevant thresholds: EF<50% for evidence of “reduced ejection fraction” and EF<35% as a common threshold for implantable cardioverter-defibrillator therapy in heart failure [16].

## 3 Results

The U-net for the trabecular model was run for 350 epochs, at which point there was <10^−4^ change in the cost function. This took 3 h on a 16-core CPU with 64 Gb of RAM and Nvidia RTX-2080Ti GPU. The final value of the cost function (categorical cross entropy) was 0.57.

### 3.1 Subjective assessment of segmentation quality

Segmentations from 1594 successive patient scans were evaluated, consisting of 29,680 individual images. The breakdown of errors by type, location, and cause is reported in Table 1, and example errors are shown in Fig. 2.

**Table 1:** Breakdown of significant segmentation errors from 1594 consecutive clinical cases

	Rounded	Trabecular	Total	p-value
Diastole	29 (2%)	32 (2%)	33 (2%)	0.7
Systole	33 (2%)	39 (2%)	39 (2%)	0.47
Base	33 (2%)	34 (2%)	34 (2%)	0.91
Mid	3 (0.2%)	4 (0.3%)	4 (0.3%)	0.7
Apex	14 (1%)	15 (1%)	14 (1%)	0.85
Extreme anatomy	6 (0.4%)	6 (0.4%)	6 (0.4%)	1
Severe artifact	4 (0.3%)	4 (0.3%)	4 (0.3%)	1

Data reflects absolute number of clinical cases with error and in brackets, percentage of cases with error out of 1594 clinical cases assessed.

In total, 184/29680 (0.6%) images were affected by significant segmentation error. Multiple errors affected some patients with clinically significant errors affecting 55/1594 (3.4%) and 60/1594 (3.6%) patients using the rounded and trabecular models, respectively. Because of the coupled approach to trabecular segmentation, all rounded segmentation errors also propagated into trabecular segmentations, but the difference was not significant. Most significant errors occurred at the LV base (66%), followed by the apex (26%) with relatively few occurring in mid cavity (8%). More errors occurred in systole (54%) than diastole. Extreme anatomy (e.g., large aneurysms) or severe artifact (e.g., extensive wrap or mis-gating) accounted for 20% of significant errors.

Interobserver agreement for segmentation error was assessed on clinical scans from 533 consecutive patients. For the rounded model 10 patients were identified with errors by both observers and 5 patients showed discordant classification, resulting in Cohen’s κ = 0.80 (95% CI: 0.62–0.97), indicating substantial agreement. For the trabecular model, 17 patients were identified with errors by both observers and 9 patients showed discordant classification, yielding Cohen’s κ = 0.78 (95% CI: 0.64–0.92), also reflecting substantial agreement.

### 3.2 Differences in volumes and function

**Fig. 3:**
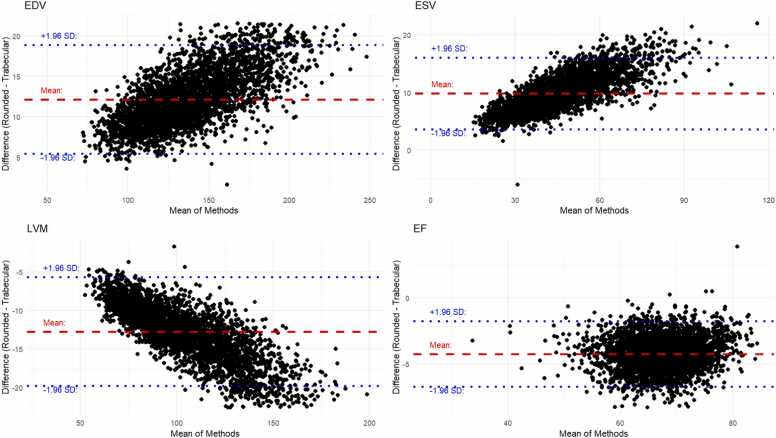
Bland-Altman plots of the difference between rounded and trabecular segmentation for left ventricle metrics on a healthy subset of the UK Biobank imaging substudy. *EDV*, end diastolic volume, *ESV*, end systolic volume, *LVM* left ventricular mass, *EF* ejection fraction

Mean values for all LV metrics for both rounded and trabecular segmentations are tabulated in Table 2. The mean LV EDV of the 4118 healthy subjects was significantly higher for rounded segmentations (female: 124 mL, male: 164 mL) than for the trabecular segmentations (female: 114 mL, male: 150 mL). As expected, LV mass was significantly higher when trabeculae and papillary muscles are included (mean: female: LVM = 90 g, male 132 g, for trabeculated vs female: 79 g & male: 117 g rounded). The Bland-Altman plots in Fig. 3 show that the difference in blood volumes and myocardial mass becomes more pronounced with higher values, but remains more consistent with EF.

**Table 2:** A comparison of LV metric values obtained by applying rounded and trabecular models to 4118 healthy subjects from the UK Biobank

	Female				Male			
	Mean	SD	Lower Limit	Upper Limit	Mean	SD	Lower Limit	Upper Limit
EDV rounded (mL)	124	20.5	83.1	165	164	28.6	107	221
EDV trabecular (mL)	114	19.1	75.5	152	150	26.4	96.7	202
ESV rounded (mL)	41.9	11.2	19.5	64.3	59.9	16.6	26.7	93
ESV trabecular (mL)	33.7	9.73	14.3	53.2	48	14.1	19.8	76.2
SV rounded (mL)	82.2	13.4	55.5	109	104	18.6	67	141
SV trabecular (mL)	80.1	13.2	53.7	106	102	18.1	65.3	138
EF rounded (%)	66.5	5.7	55.2	77.8	63.8	6.3	51.3	76.3
EF trabecular (%)	70.6	5.7	59.3	81.9	68.2	6.1	56	80.3
LVM rounded (g)	79.2	12.4	54.3	104	117	20	77.1	157
LVM trabecular (g)	90	14.4	61.3	119	132	22.9	86.5	178
EDVi rounded (mL/m²)	72.7	10.4	51.9	93.4	83.1	13.4	56.3	110
EDVi trabecular (mL/m²)	66.6	9.82	47	86.3	75.8	12.5	50.8	101
ESVi rounded (mL/m²)	24.5	6.05	12.4	36.6	30.3	8.03	14.2	46.4
ESVi trabecular (mL/m²)	19.7	5.35	9.03	30.4	24.3	6.92	10.5	38.1
SVi rounded (mL/m²)	48.2	6.97	34.2	62.1	52.8	8.84	35.1	70.5
SVi trabecular (mL/m²)	46.9	6.86	33.2	60.6	51.5	8.61	34.2	68.7
LVMi rounded (g/m²)	46.3	5.83	34.7	58	59.2	8.43	42.3	76
LVMi trabecular (g/m²)	52.6	6.77	39.1	66.2	66.9	9.78	47.3	86.5

LL is the lower limit of the normal reference interval and UL is the upper limit. *EDV* end diastolic volume, *ESV* end systolic volume, *SV* stroke volume, *EF* ejection fraction, *LVM* left ventricular mass, *EDVi* indexed end-diastolic volume, *ESVi* indexed end systolic volume, *SVi* indexed stroke volume, *LVMi* indexed left ventricular mass

Data provided reflects the summary statistic in the second row for each sex (top row).

A significant difference exists for EF, with a mean value of 66.5% and 70.6% for rounded segmentations and 63.8% and 68.2% for trabecular segmentation in females and males respectively, despite similar stroke volumes. The difference is because the denominator, EDV, is significantly lower for trabecular segmentation—see Fig. 1 for an illustration of this effect.

### 3.3 Scan-rescan precision

**Fig. 4:**
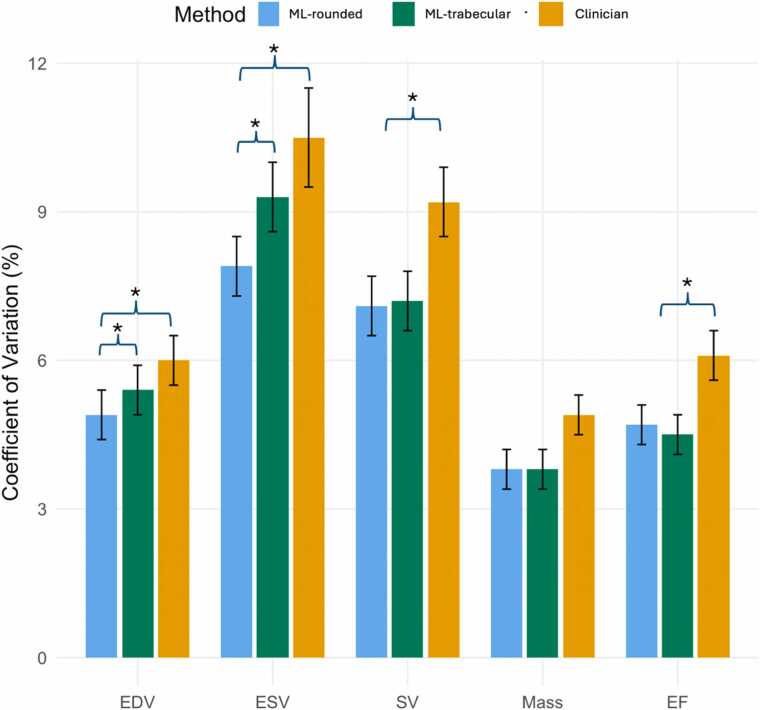
Scan-rescan precision of left ventricular volumes and function from 109 subjects with repeat scans within 30 days. The coefficient of variation is reported (with standard deviation in brackets) for each metric for rounded segmentations by ML (blue), trabecular by ML (green) and rounded by clinician (orange). The error bars represent ±1 standard deviation. * denotes a statistically significant difference after Bonferroni correction for multiple testing [p = 0.0033 (0.05/15)]. For end diastolic volume and end systolic volume, only ML rounded segmentation had significantly better precision than clinician, for stroke volume and LV ejection fraction, both ML rounded and ML trabecular had better precision. *ML* machine-learning, *LV* left ventricle

**Fig. 5:**
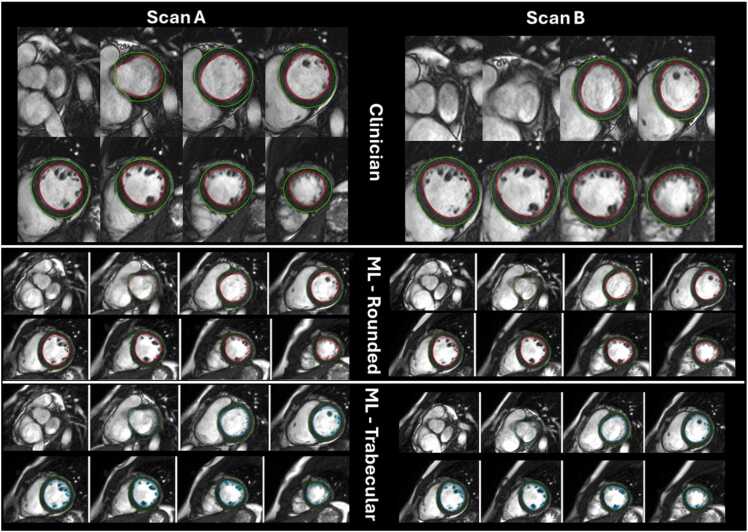
Segmentations on a healthy volunteer test-retest pair in diastole. Left: Scan A; Right: Scan B. Top row: clinician segmentation (rounded); Middle row: machine learning segmentation (rounded); Bottom row: machine learning (trabecular). Note only the first 8 slices are drawn here — all slices including systole are illustrated in the supplement. A dashed line on the machine learning segmentation signifies that the valve plane intersects the contour and only part of this volume is included

Scan-rescan precision results are presented in Fig. 4 and Table 3a, Table 3b. Exemplar scan-rescan segmentations are shown in Fig. 5 and Supplementary Figure 2. There was no significant difference between the coefficients of variation (CoV) of rounded and trabecular model for SV, mass, and EF. The rounded model had better precision for EDV and ESV (Table 3a).

**Table 3a:** Test-retest precision comparison of ML trabecular vs ML rounded segmentation

	ML Trabecular							ML Rounded							ML Trabecular vs Rounded
	CoV %	CoV 95%CI low	CoV 95%CI high	CoV SD	SEM	MDC	ICC	CoV %	CoV 95%CI low	CoV 95%CI high	CoV SD	SEM	MDC	ICC	p value for CoV difference
EDV	5.4	4.3	6.4	0.5	7.6	21.1	0.98	4.9	4.0	5.8	0.5	7.4	20.6	0.98	0.0002
ESV	9.3	7.8	10.7	0.7	5.1	14.1	0.98	7.9	6.6	9.1	0.6	4.9	13.7	0.99	<0.001
SV	7.2	5.9	8.4	0.6	6.5	18.0	0.93	7.1	5.9	8.4	0.6	6.3	17.5	0.93	0.77
Mass	3.8	3.1	4.5	0.4	5.3	14.6	0.99	3.8	3.0	4.5	0.4	4.9	13.5	0.99	0.70
EF	4.5	3.8	5.2	0.4	2.7	7.5	0.95	4.7	3.9	5.3	0.4	2.6	7.1	0.95	0.43

*CoV* Co-efficient of variation with 95% lower and higher limits, *SD* standard deviation, *SEM* standard error of measurement, *MDC* minimal detectable change, *ICC* intraclass correlation co-efficient, *EDV* end diastolic volume, *ESV* end systolic volume, *SV* stroke volume, *EF* ejection fraction. A Bonferroni correction would require a value of p = 0.0033 (0.05/15) to declare statistical significance at a nominal type I error rate of 0.05

Data reflects each precision metric given in the second row for ML Trabecular vs ML rounded segmentation (top row).

**Table 3b:** Test-retest precision comparison of ML trabecular and ML rounded segmentation vs Clinicians

	Clinician								
	CoV %	CoV 95%CI low	CoV 95%CI high	CoV SD	SEM	MDC	ICC	ML Trabecular vs Clinician p value for CoV difference	ML Rounded vs Clinician p value for CoV difference
EDV	6.0	4.9	7.0	0.5	8.7	24.2	0.97	0.119	**0.002**
ESV	10.5	8.6	12.4	1.0	6.3	17.4	0.98	0.14	**0.001**
SV	9.2	7.8	10.6	0.7	7.9	21.9	0.88	**0.001**	**0.003**
Mass	4.9	4.1	5.6	0.4	6.8	18.8	0.98	0.031	0.028
EF	6.1	5.2	7.1	0.5	3.4	9.4	0.91	**0.0008**	**0.004**

*CoV* Co-efficient of variation with 95% lower and higher limits, *SD* standard deviation, *SEM* standard error of measurement, *MDC* minimal detectable change, *ICC* intraclass correlation co-efficient, *EDV* end diastolic volume, *ESV* end systolic volume, *SV* stroke volume, *EF* ejection fraction. A Bonferroni correction would require a value of p = 0.0033 (0.05/15) to declare statistical significance at a nominal type I error rate of 0.05

Both ML trabecular and rounded models had better precision than clinicians for stroke volume and EF (Table 3b). ML models did not have better precision than clinicians for LV mass. The improved MDC afforded by the ML trabecular model over clinician translates to fewer subjects needed to achieve the same statistical power in a clinical trial. For an EF endpoint, 37% fewer subjects would be needed (alpha 0.05, power 0.9).

### 3.4 Clinical outcome and EF thresholds

All LV volumetric parameters were predictive of outcome. Rounded contours had slightly lower AIC and higher χ² for EDVi, ESVi and EF, whereas trabecular contours showed slightly lower AIC and higher χ² for SVi. There was no difference in prediction for LVMi. Differences in discrimination (C-Indices) between rounded and trabecular models were marginal across all metrics **(**Table 4**)**.

**Table 4:** ML rounded vs trabecular LV parameters Cox Proportional Hazard’s models for prediction of outcome (all-cause mortality or hospitalization with heart failure)

	Rounded						Trabecular						Rounded vs Trabecular		
Metric	β	HR (95% CI)	p value	χ²	C-index	AIC	β	HR (95% CI)	p value	χ²	C-index	AIC	ΔC-index	Δχ²	p value
EDVi	0.0104	1.010 (1.007–1.014)	<0.001	33	0.59	3777	0.0091	1.009 (1.005–1.013)	<0.001	20	0.568	3789	0.022	13	<0.001
ESVi	0.0123	1.012 (1.009–1.016)	<0.001	57	0.631	3753	0.0124	1.012 (1.009–1.016)	<0.001	48	0.616	3762	0.015	9	0.0026
SVi	-0.042	0.959(0.947-0.970)	<0.001	49	0.618	3760	-0.0468	0.954(0.943-0.966)	<0.001	62	0.636	3748	-0.018	13	<0.001
LVMi	0.023	1.023 (1.019–1.028)	<0.001	85	0.668	3725	0.0199	1.020 (1.016–1.024)	<0.001	86	0.671	3724	−0.003	1	0.23
EF	−0.034	0.967 (0.960–0.973)	<0.001	88	0.657	3722	−0.031	0.969 (0.963–0.976)	<0.001	81	0.649	3729	0.008	7	0.0077

*EDVi* indexed end-diastolic volume, *ESVi* indexed end systolic volume, *SVi* stroke volume indexed, *LVMi* LV Mass indexed, *EF* ejection fraction

Data reflects performance metric provided in the second. Hazard ratios are provided with 95% confidence intervals in brackets.

For EF <35%, rounded contours classified 117 patients and trabecular contours classified 119 patients as below threshold; 44/1215 (4%) patients were classified differently between the 2 methods. For EF <50%, rounded contours classified 309 patients and trabecular contours classified 302 patients as below threshold; 71/1215 (6%) patients were classified differently between the 2 methods.

## 4 Discussion

By building robust, generalizable and fully automated segmentation, we have been able to compare rounded with trabecular segmentations at scale. Our main findings are (1) when trained with appropriate data, ML has potential to be generalizable (any disease, any scanner) and robust (errors in <1% of images) (2) trabecular and rounded segmentations produce different values for all LV metrics, which could impact guideline-directed care (3) differences in prognostication were marginal (4) there is no significant difference between the test-retest precision of trabecular and rounded segmentations for key LV metrics: SV, LV Mass and EF (5) both ML models have better precision than a clinician for ejection fraction, which could have significant implications for clinical trials and drug development.

There is a significant difference in volumes produced by the two segmentation approaches, with rounded segmentations producing higher blood volumes and trabecular segmentation generating higher LV mass. This is an expected result and a well-recognized phenomenon as also demonstrated in a large international CMR consortium of healthy individuals [17]. The Society for Cardiovascular Magnetic Resonance (SCMR) now provides separate reference ranges for rounded and trabecular segmentations [11]. However, many disease definitions and management guidelines use a one-method-for-all approach where the method of segmentation is not specified [18]. Examples include arrhythmogenic right ventricular cardiomyopathy [19], heart failure with reduced ejection fraction [16], and valvular disease [20]. In healthy subjects, we found the mean EF was 4% higher with trabecular segmentation. Decisions regarding implantable cardioverter-defibrillator implantation and classification of heart failure phenotypes (e.g., heart failure with reduced versus preserved EF) are based on fixed EF thresholds irrespective of segmentation method [16]. However, given the systematic differences in EF between techniques, these thresholds may require adjustment depending on the method used. In our clinical cohort, 9.4% of patients were classified differently at clinically relevant EF thresholds when comparing trabecular versus rounded segmentation, highlighting potential implications for prognostication and management.

There was no significant difference in scan-rescan precision between the two segmentation approaches for SV, LVM, and EF. *A priori*, we expected rounded segmentations to be more reproducible since drawing a simple circle has fewer degrees of freedom than creating multiple detailed annotations of several small structures. Furthermore, we expected the trabecular segmentations to be more sensitive to flow-induced artifacts in the blood pool, but few examples of this was seen, illustrating the robustness of ML segmentation tools. Both ML segmentation methods had better reproducibility than a clinical expert for EF, which has positive implications for clinical trials. For example, if the ML was adopted for a trial with an EF endpoint, potentially an estimated 37% fewer subjects would be needed to achieve the same statistical power. Another trabecular segmentation technique using a U-net approach (DenseNet) has demonstrated reproducible segmentation of cardiac trabeculation [6] and has been applied to the UK Biobank to show important relationships of trabecular/papillary muscle mass to comorbidities, exposures, demographics, and ethnicity [17], [21]. Here, we have developed trabecular automated segmentation further from a larger training dataset (∼7X more subjects) and examine relationships with prognostication.

Segmentations in 99.4% of images (96% of patients) were free of a clinically significant error. In those with a significant error, most mis-segmentations were obvious and easy to correct. Although we fully endorse the use of automated ML tools for cardiac segmentation, we also recognize and advocate that clinical oversight remains a vital part of the cardiac MR analysis workflow.

We emphasize, however, that none of the segmentations in either the scan-rescan precision or prognosis evaluation were corrected, meaning that even with uncorrected errors, the automated tool had superior precision to an experienced clinician.

All differences in prognostication between ML-rounded and ML-trabecular segmentation as measured by model fit statistics (AIC, C-index, χ²) were marginal meaning there is no clinically meaningful difference in prognostication between trabecular and rounded segmentation, though further work is warranted in diseases with diverse morphology, such as hypertrophic cardiomyopathy and left ventricular hypertrophy.

Given the absence of substantial differences in error rate, the propagation of similar error types between methods, and no clinically meaningful differences in prognostication, use of trabecular segmentation appears feasible. Furthermore, adopting trabecular segmentation may improve consistency with EF derived from cardiac CT which can reliably measure EF using trabecular contours with minimal radiation dose [22]. Clinical workflows adopting this ML segmentation benefit from dramatically improving speed and reducing cost of acquiring clinically important LV metrics. Furthermore, adopting ML-derived LV metrics, overcoming clinician heterogeneity, may improve our ability to detect serial change in function, standardize measurements across scanners and international boundaries, and detect subclinical disease. One of the challenges of improving patient care with new methods is clinical translation. We have achieved this by implementing the method inline on CMR scanners, using the Gadgetron framework. This allows ML analysis to be performed whilst the patient is still in the scanner and results are ready by the time that the study is opened for reporting. Both rounded and trabecular segmentations have already been applied to thousands of patient studies.

## 5 Limitations

There are limitations to this work. First, this was a retrospective analysis. Second, we have compared segmentation approaches using a specific ML tool. Although we expect the results reported here to generalize to all segmentation tools (and to manual clinical annotation), further work is needed to prove this using different annotators (clinicians or ML). The automated trabecular segmentation model is not open-source, and therefore, this reduces reproducibility in the community. Trabeculae are small structures that are therefore affected by partial voluming in CMR and all segmentation techniques relies on a degree of subjectivity which may affect expert labels used to train the model. Given the inherent subjectivity of contouring, we selected expert reader-rounded contours as the reference, as this approach is expected to provide the highest reproducibility among clinicians. Furthermore, the segmentation approach uses the mitral annular plane to delineate the base of the left ventricle, which we previously found improves test-retest precision [4]. This may, however, exclude part of the left ventricular outflow tract, resulting in left ventricular blood volumes being slightly underestimated. The Bland-Altman plots show a larger discrepancy between trabecular and rounded volumes when the absolute volumes are bigger (Fig. 3), which will introduce a proportional bias. No data harmonization for different scanners was performed in this study, so estimated cardiac volumes may differ between different models and field strengths. The sample-size implications derived from minimal detectable change and CoV estimates should be regarded as illustrative rather than prescriptive, since they assume identical variance structures and endpoint behavior across imaging sites and trial designs, which may not hold in practice. Prognostic comparisons were unadjusted and should be interpreted as exploratory differences in model-fit statistics rather than evidence of clinical superiority of either segmentation approach.

## 6 Conclusion

We developed an automated method for annotating papillary muscles and trabeculae from cardiac MR images with low error rates. We found higher precision than clinicians in ejection fraction. There was similar precision and prognostication to an ML rounded model with similarly low error rates. Findings support the feasibility of automated trabecular segmentation in clinical care and clinical trials.

## Funding

GJ is funded by an NIHR Clinical Lectureship. RhHD was funded by the British Heart Foundation (BHF) Accelerator Award (AA/18/6/34223) and University College London Hospitals (UCLH) NIHR Biomedical Research Centre (BRC). J.C.M., C.M. and T.A.T. were directly and indirectly supported by the University College London Hospitals (UCLH) and Barts NIHR Biomedical Research Centres and BHF Accelerator Award (AA/18/6/34223). TAT is funded by a BHF Intermediate Research Fellowship (FS/19/35/34374). M.F. is supported by a BHF Intermediate Research Fellowship (FS/18/21/33447).

## Author contributions

**George Joy:** Writing – review & editing, Writing – original draft, Validation, Software, Methodology, Investigation, Formal analysis. **James C. Moon:** Writing – original draft, Methodology, Investigation, Funding acquisition, Conceptualization. **Karan Punjabi:** Writing – original draft. **Mohammed Alzahir:** Writing – review & editing, Validation, Data curation. **Jessica Artico:** Writing – review & editing, Validation, Data curation. **Hunain Shiwani:** Writing – review & editing, Data curation. **Iain Pierce:** Writing – review & editing, Data curation. **Anish Bhuva:** Writing – review & editing, Methodology, Data curation. **Dhruv Thakur:** Writing – review & editing, Formal analysis, Data curation. **Hui Xue:** Writing – review & editing, Software, Methodology. **Peter Kellman:** Writing – review & editing, Validation, Investigation, Formal analysis. **Erik Schelbert:** Writing – review & editing, Investigation, Data curation. **Thomas A. Treibel:** Writing – review & editing, Methodology, Data curation. **Charlotte Manisty:** Writing – review & editing, Methodology, Data curation. **Rhodri H. Davies:** Writing – original draft, Visualization, Validation, Software, Project administration, Methodology, Investigation, Formal analysis, Conceptualization.

## Declaration of Competing Interest

The authors declare the following financial interests/personal relationships which may be considered as potential competing interests: Rhodri Davies reports a relationship with Mycardium that includes consulting or advisory. JCM, HS, TAT, CM, and RhHD own shares in Mycardium.AI. GJ is a consultant for Mycardium.AI. Mycardium.AI holds a licensing agreement with UCL to commercialise the work described here. Other authors declare that they have no known competing financial interests or personal relationships that could have appeared to influence the work reported in this paper.
