# Supplementary material for: Precision, prognosis, and clinical performance of rounded and trabecular segmentation of cine cardiovascular magnetic resonance
Source: J Cardiovasc Magn Reson. 2025 Nov 25;28(1):102014. doi: 10.1016/j.jocmr.2025.102014 (PMC12811440; doi:10.1016/j.jocmr.2025.102014)
Supplement: Supplementary file 1 — Supplementary material [file mmc1.docx]

SUPPLEMENT

| Age, years (median [IQR]) | 57 [45, 66] |
| --- | --- |
| Male, no (%) | 741 (58%) |
| Ethnicity, no (%) | 1114 (87%) white |
|  | 130 (10%) Afro-Carribean |
|  | 9 (<1%) Asian |
|  | 2 (<1%) Hispanic |
| BMI, Kg/m^2, median [IQR] | 28.7 [24.9, 34.0] |
|  |  |
| No past medical history, no (%) | 204 (16%) |
| Diabetes, no (%) | 234 (18%) |
| Hyperlipidaemia, no (%) | 491 (38%) |
| Current or ex-smoker, no (%) | 562 (44%) |
|  |  |
| Atrial fibrillation, no (%) | 128 (10%) |
| Pre-existing diagnosis of heart failure, no (%) | 255 (20%) |
| Previous MI, no (%) | 208 (26%) |
| Known coronary artery disease, no % | 234 (18%) |
| ICD in situ, no (%) | 135 (10%) |

**Supplementary Table 1: clinical details of the prognosis dataset.**


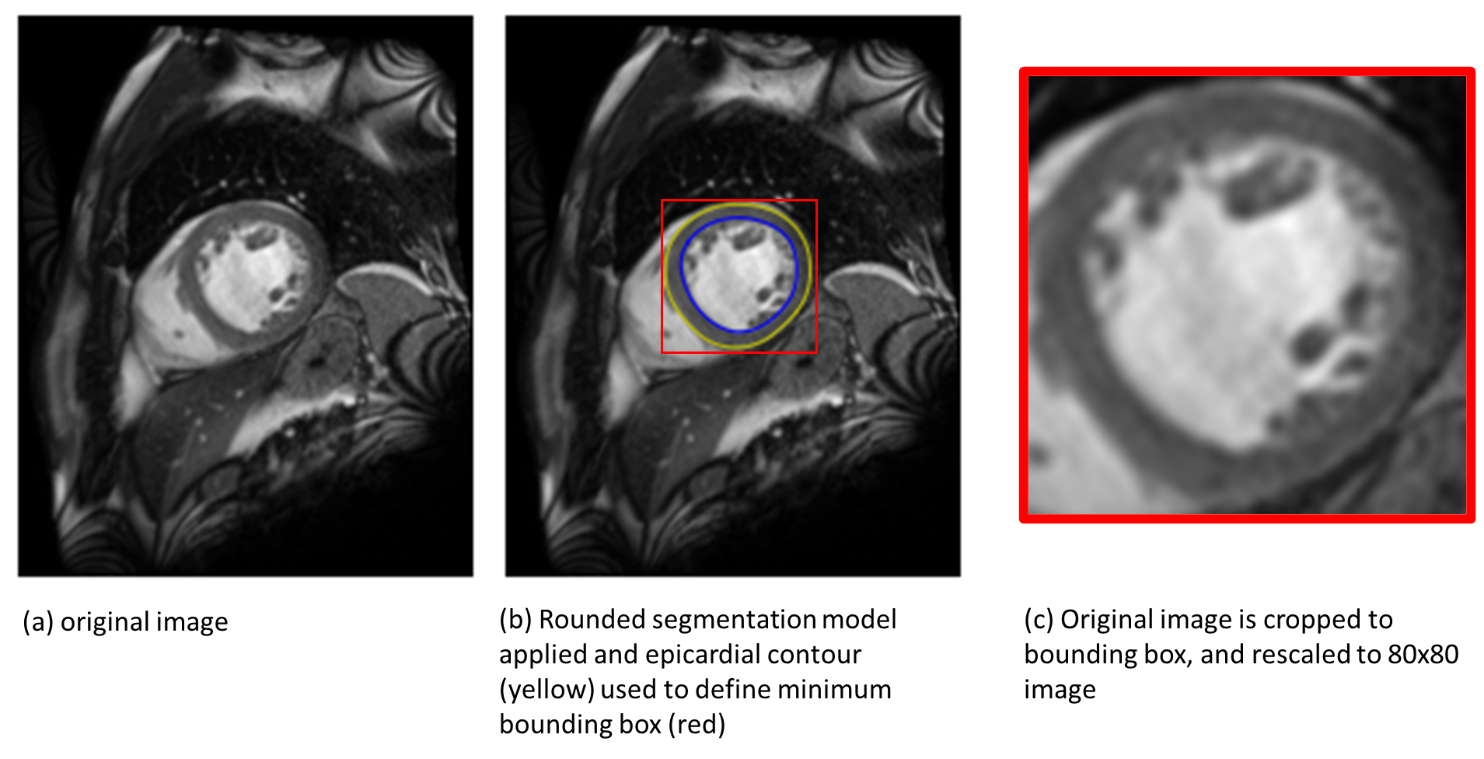


**Supplementary Figure 1: illustration of pre-processing for training of Trabecular model.** The rounded model is first applied to the image to identify the epicardial border (yellow contour), which defines a minimum bounding box (red box). The original image is cropped to the bounding box and rescaled to 80x80 pixels.


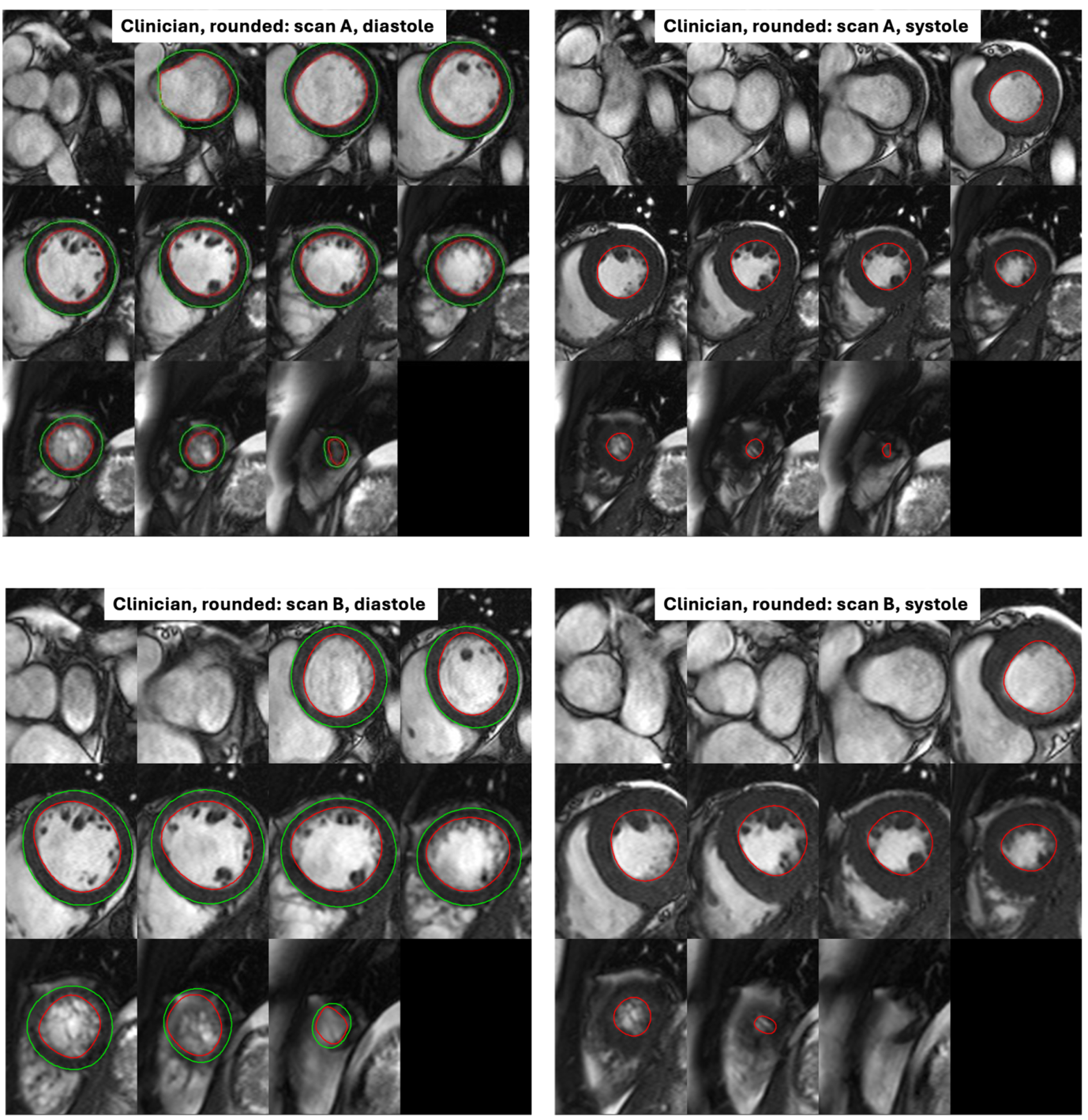


**Supplementary Figure 2a Segmentations on a healthy volunteer test-retest pair in diastole and systole using clinician segmentation**


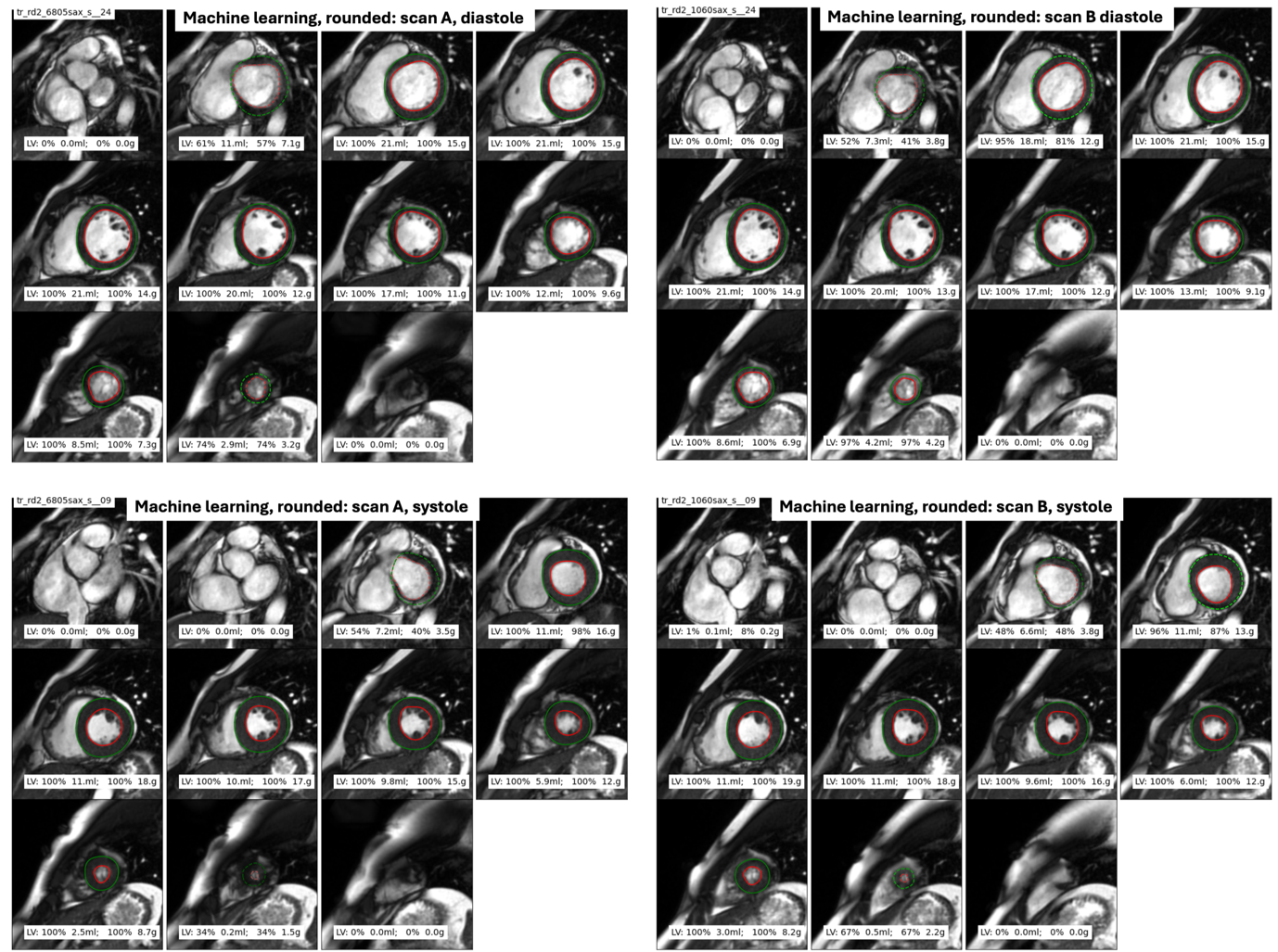


**Supplementary Figure 2b Segmentations on a healthy volunteer test-retest pair in diastole and systole using ML rounded segmentation**


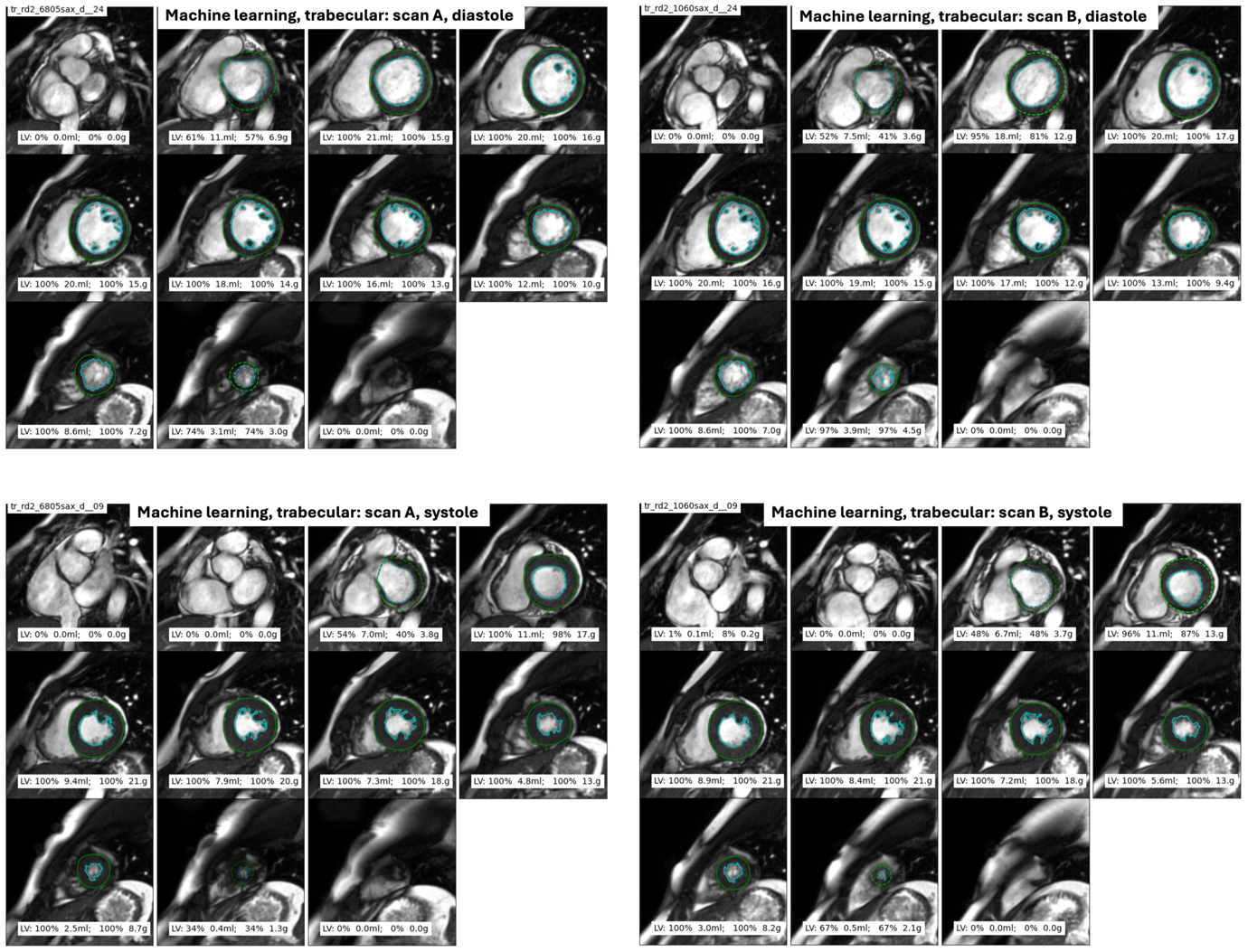


**Supplementary Figure 2c Segmentations on a healthy volunteer test-retest pair in diastole and systole using ML trabecular segmentation**
